# Supplementary material for: Chemical-induced phase transition and global conformational reorganization of chromatin
Source: Nat Commun. 2023 Sep 9;14:5556. doi: 10.1038/s41467-023-41340-4 (PMC10492836; doi:10.1038/s41467-023-41340-4)
Supplement: Supplementary file 1 — Supplementary Information [file 41467_2023_41340_MOESM1_ESM.pdf]

Supplementary Information

Supplementary Methods

Supplementary Figures

Supplementary Fig. 1. The correlation between the cell cycle adriamycin-induced condensates.
Supplementary Fig. 2. Adriamycin-induced chromatin condensation is independent of apoptosis.
Supplementary Fig. 3. Quantification of the clustering level of chromatin upon the treatment of different drugs.
Supplementary Fig. 4. Synthesis and characterization of Texas Red-Cisplatin.
Supplementary Fig. 5. Adriamycin-induced chromatin condensates are irrelevant to transcription- or DNA damage-associated chromatin condensates.
Supplementary Fig. 6. Adriamycin-induced chromatin condensation is independent of TOP2 isozyme.
Supplementary Fig. 7. The gain and loss of chromatin accessibility upon adriamycin treatment.
Supplementary Fig. 8. Transcriptomic comparison of etoposide and adriamycin treatment on U2OS cells.
Supplementary Fig. 9. Changes in TAD organization contribute to gene expression shifts.
Supplementary Fig. 10. The C-terminal of histone H1 interacts and forms condensates with adriamycin.
Supplementary Fig. 11. The gating strategy and uncropped blots/gels in Supplementary Figures.

Supplementary Data

Supplementary Data 1. List of differentially expressed genes upon etoposide or adriamycin treatment.
Supplementary Data 2. Association analysis of differentially expressed genes and gain/loss of ATAC-seq peaks.
Supplementary Data 3. List of genes associated with compartment A/B switch.
Supplementary Data 4. List of differentially expressed transposable elements upon etoposide or adriamycin treatment.

For Supplementary Data 1-3, two-tailed P values were calculated by Wald test and the resulting P values were adjusted (Padj) by Benjamini and Hochberg's method for controlling the false discovery rate. Padj<0.01 and $|\log_2(\text{foldchange})| > 1$ were set as the threshold for significantly differential expression.

Supplementary Movies

Supplementary Movie 1. Live imaging of adriamycin condensate formation in mouse embryonic fibroblasts
Supplementary Movie 2. FRAP analysis on adriamycin condensates in MEFs
Supplementary Movie 3. *In vitro* condensation of native chromatin upon adriamycin treatment
Supplementary Movie 4. *In vitro* formation of H1-adriamycin condensates
Supplementary Movie 5. FRAP analysis on H1-adriamycin condensates *in vitro*

Supplementary Methods

Synthesis of Texas Red-Cisplatin

K_2PtCl_4 (98%) and tert-butyl(2,3-diaminopropyl)carbamate (95%) were purchased from Bidepharm; Texas Red^{TM}-X succinimidyl ester (mixed isomers) was purchased from ThermoFisher Scientific; Hydrochloric acid (HCl, ~36.5 wt %, 12 mol/L) (AR), triethylamine (≥ 99.0 %) and acetonitrile (MeCN, ≥ 99.8 %) were purchased from Sinopharm Chemical Reagent Co., Ltd. Commercially available substrates were used without further purification unless otherwise noted. All manipulations of reagents were carried out under N_2 atmosphere. Texas Red-Cisplatin was synthesized based on previous report with slight modifications [1]. Reactions were carried out in capped cylindrical vials equipped with a magnetic stir bar and heated in a metal heating block. The composition and molecular characterization were analyzed by HPLC-MS and NMR spectra. The detailed synthesis procedure and the ^{1}H NMR results are available upon request.

Purification of His-tagged H1 and H1-CFP

pET28 plasmids encoding His–tagged H1 or H1-CFP (full length and fragments) were transformed into BL21(DE3) *E. coli*. Cells were grown in 1 L of LB media supplemented with 50 µg/ml kanamycin at 37 °C until the OD_{600} reached 0.6. Protein expression was induced with 0.5 mM IPTG at 16 °C overnight. Cells were collected by centrifugation, resuspended in lysis buffer (20 mM Tris, pH 7.5, 50 mM NaCl, 10 mM imidazole, 0.5 mM $NiCl_2$), and lysed by either sonication or the French pressure cell press. The supernatant was isolated by centrifugation for 30 min at 4 °C at 24,000 g and purified by Ni-NTA affinity chromatography (GE Healthcare Life Sciences, 17-5318-02) on a gravity column. Ni-NTA resin was washed with 20 column volumes of lysis buffer and then 10 column volumes of wash buffer (15 mM Tris, pH 7.5, 500 mM NaCl, 20 mM imidazole) before elution with elution buffer (15 mM Tris, pH 7.5, 500 mM NaCl, 300 mM imidazole). Glycerol was added to the purified protein to a final concentration of 5%, and the protein samples were aliquoted and stored at –80 °C.

Coomassie Blue Gel Staining

The protein samples were analyzed on 4-20% SDS-PAGE gel (Yeasen, 36256ES10). Staining was performed by covering gels with Coomassie Blue SuperFast Staining Solution (BeyoBlue, P0017FFT) and incubated at room temperature for 60 minutes with gentle agitation. Gels were rinsed and washed with ultrapure water overnight. Uncropped and unprocessed scans of gels are provided in the Source Data file or in the Supplementary Information.

Western blot

After drugs treatment, cells were lysed in sample buffer containing 2% SDS, 7.8% glycerol, 10 mM Tris.Cl pH 6.8, 1.55% DTT, and 0.01% bromophenol blue. After boiling, proteins were separated by SDS-PAGE (GenScript, M00657) and transferred to PVDF membranes. After blocking in 5% non-fat milk in TBS containing 0.1% Tween-20 (TBST), membranes were probed with primary antibodies followed by horseradish peroxidase (HRP)-conjugated secondary antibodies. The signal was visualized by Omni-ECL kit (EpiZyme, SQ202). The primary antibodies used are: γ-H2AX (ABclonal, AP0099), 1:1000; p53 (Santa Cruz Biotech, sc-126), 1:1000; β-Actin (ABclonal, AC026), 1:1000. The secondary antibodies used are: HRP goat anti-mouse IgG (ABclonal, AS003) and HRP goat

anti-rabbit IgG (ABclonal, AS014) (1:5000). Uncropped and unprocessed scans of blots and gels are provided in the Supplementary Information.

Apoptosis and FACs analysis

For FACs analysis, 2 x 10^5 U2OS cells were treated with 15 μM sorafenib (MCE；HY-10201) or TNFα + CHX (TNFα 5 ng/ml [Peprotech, 300-01A] and cycloheximide 5 μg/ml [MCE, HY-12320]) for 16h. Attached cells were digested with accutase and combined with suspended cells, washed twice with cold PBS, and resuspended in 1X binding buffer from Annexin V-EGFP/PI apoptosis assay kit (Yeasen, 40303ES20). 100 μL of 1~5 x 10^6/ml cell suspension was mixed with 5 μL Annexin V-EGFP and incubated in RT for 5 min. After adding 10 μL PI staining solution, 400 μL PBS was added before FACs analysis on Fortessa (BD Biosciences).

Preparation of RNA-Seq library

Total RNA was extracted using Trizol (Vazyme, Cat.R401-01). Total RNA was used as input material for the RNA sample preparations. Briefly, mRNA was purified from total RNA by using poly-T oligo-attached magnetic beads. Fragmentation was carried out using divalent cations under elevated temperature in First Strand Synthesis Reaction Buffer (5X). First strand cDNA was synthesized using random hexamer primer and M-MuLV Reverse Transcriptase, then use RNase H to degrade the RNA. Second strand cDNA synthesis was subsequently performed using DNA Polymerase I and dNTP. Remaining overhangs were converted into blunt ends via exonuclease/polymerase activities. After adenylation of 3' ends of DNA fragments, Adaptor with hairpin loop structure were ligated to prepare for hybridization. In order to select cDNA fragments of preferentially 370~420 bp in length, the library fragments were purified with AMPure XP system (Beckman Coulter, Beverly, USA). Then PCR amplification, the PCR product was purified by AMPure XP beads, and the library was finally obtained.

In order to ensure the quality of the library, the library needs to be tested. After the construction of the library, the library was initially quantified by Qubit2.0 Fluorometer, then diluted to 1.5ng/ul, and the insert size of the library is detected by Agilent 2100 bioanalyzer. After insert size meets the expectation, qRT-PCR is used to accurately quantify the effective concentration of the library (the effective concentration of the library is higher than that of 2nM) to ensure the quality of the library.

Hi-C library construction.

Hi-C libraries were prepared following previously published in situ Hi-C protocols with minor modifications (Rao, Huntley et al. 2014, Belaghzal, Dekker et al. 2017, Zuo, Chen et al. 2021). For each Hi-C experiment, 1 × 106 U2OS cells were fixed with 920 μL 1% v/v formaldehyde in 1× HBSS at RT for 10 min. After fixation, 80 μl 2.5 M glycine was added to each reaction. Cells were then incubated at RT for 5 min and on ice for at least 15 min to quench the crosslinking reaction. The fixed U2SO cells were collected by centrifugation at 800 × g for 5 min at 4 °C and resuspended with 200 μl ice-cold lysis buffer (10 mM Tris-HCl pH 8.0, 10 mM NaCl, 0.2% Igepal CA-630) with protease inhibitors. All cells were incubated on ice for 30 min and were centrifuged for 5 min at 3000 × g at 4 °C to isolate nuclei. The nuclei pellets were washed once with 100 μl 1× NEB buffer 3.1, resuspended in 2 μl 10× NEB buffer 3.1, 14 μl ddH2O, and 4 μl of 0.5% SDS, and incubated at 65 °C for 5 min to open up chromatin. All samples were then incubated at 37 °C for 15 min in 1% Triton X-100 (Sigma, 93443) to quench SDS.

After the treatment, 12 μl of 10× NEBuffer 3.1 was added to each sample. The chromatin was then digested with DpnII (NEB, 40 μl of 10000 units/ml) overnight at 37 °C in a ThermoMixer with interval shaking (shake at 950 rpm for 10 s with 5 min intervals). Following the digestion, the samples were incubated at 65 °C for 20 min to inactivate the restriction enzyme. 60 μl biotin Fill-in Mix (2μl ddH2O, 6 μl 10× NEB 3.1, 1.5 μl 10 mM dCTP, 1.5 μl 10 mM dGTP, 1.5 μl 10 mM dTTP, 37.5 μl 0.4mM biotin-14-dATP (Life Technologies, 19524-016), 10 μl 5 U/μl DNA polymerase I Klenow) was then added to each sample. The samples were incubated at 37 °C for 1 h in a ThermoMixer with interval shaking. Following the fill-in, 665 μl ligation mix (242 μl ddH2O, 240 μl 5× ligation buffer (Invitrogen), 120 μl 10% Triton X-100, 12 μl 10 mg/ml BSA, 50 μl T4 DNA ligase (Invitrogen)) was then added to each sample and these samples were incubated at 16 °C for 4 hours in a ThermoMixer with interval shaking. After ligation, 25 μl 20 mg/ml proteinase K (NEB, P8102) was added to each sample, and all samples were incubated at 65 °C overnight to reverse the crosslinking. The purified DNA was extracted using phenol-chloroform-isoamyl- alcohol, dissolved in 130 μl 1× Tris buffer, and fragmented using Covaris M220 (peak incident power, 50 W; duty factor, 20%; cycles per burst, 200; treatment time, 70 s) to 300-500 bp. Following fragmentation, 50 μl Dynabeads MyOne Streptavidin T1 beads (Life Technologies, 65602) were added to each DNA sample to purify biotinylated DNA fragments. All samples were incubated at RT for 15 min with rotation to bind biotinylated DNA to the streptavidin beads. Beads were washed twice with 100 μl 1×Tween washing buffer (TWB, 5 mM Tris-HCl (pH 7.5); 0.5 mM EDTA; 1 M NaCl; 0.05% Tween 20) on a Thermomixer at 55 °C for 2 min with mixing and resuspended in 50 μl Milli-Q water. The purified DNA was then end-repaired, A-tailed, and adaptor-ligated using Hieff NGS® MaxUp II DNA Library Prep Kit for Illumina (Yeasen, 12200ES08). Samples were amplified with Phusion High-Fidelity DNA Polymerase (Thermo, F-530L) for 10-13 cycles, and selected 300-500bp fragments with AMPure XP beads (Beckman Coulter, A63881). All Hi-C libraries were eluted from beads with 25 μl of 1× Tris buffer and sequenced on Illumina Nova Seq 6000 platform at PE150 mode by Berry Genomics.

ATAC-seq library preparation

ATAC-seq libraries were generated from approximately 100,000 cell nuclei with the TruePrep DNA Library Prep Kit V2 for Illumina (Vazyme TD501) following a standard protocol. Briefly, 100,000 cells were lysed in 50 μl pre-cold ATAC lysis buffer [10mM Tris-HCl (pH 7.4), 10mM NaCl, 3mM $MgCl_2$, and 0.1% (v/v) Igepal CA-630], and incubated on ice for 10 min. Cells were then centrifuged at 500g at 4°C for 5 min to pellet nuclei. Pelleted nuclei were resuspended in 50 μl transposition mix (10 μl of 5×TTBL, 5 μl of TTE Mix V50, and 35 μl of double-distilled water), and then incubated at 37°C for 30 min. The reactions were purified with VAHTS DNA Clean Beads and PCR-amplified for 15 cycles. The final libraries were sequenced on Illumina Nova Seq 6000 platform at PE150 mode.

Quantitative real-time PCR analysis

Total RNA was extracted with RNA Isolator (Vazyme #R401-01). Equivalent amounts of RNA were reverse-transcribed using the HiScript II Q RT SuperMix (Vazyme #R223-01) according to manufacturer's instructions. cDNAs were then used as templates for each real-time PCR reaction using ChamQ Universal SYBR qPCR Master Mix (Vazyme, #Q711-02) on QuantStudio 7 Flex Real-Time PCR system (Life Technologies). The expression level (relative quantity, RQ) of target mRNA was determined using the ΔΔCt method with GAPDH or β-actin as the internal control with three replications. The sequences of gene-specific primers (GENEWIZ, Shanghai)are: *hTop2b*-F:

GTGAGCTGGAGGCACTCG; *hTop2b*-R: AGGTCAGTGCCCCGTTG; *hGapdh*-F: GTCTCCTCTGACTTCAACAGCG; *hGapdh*-R: ACCACCCTGTTGCTGTAGCCAA.

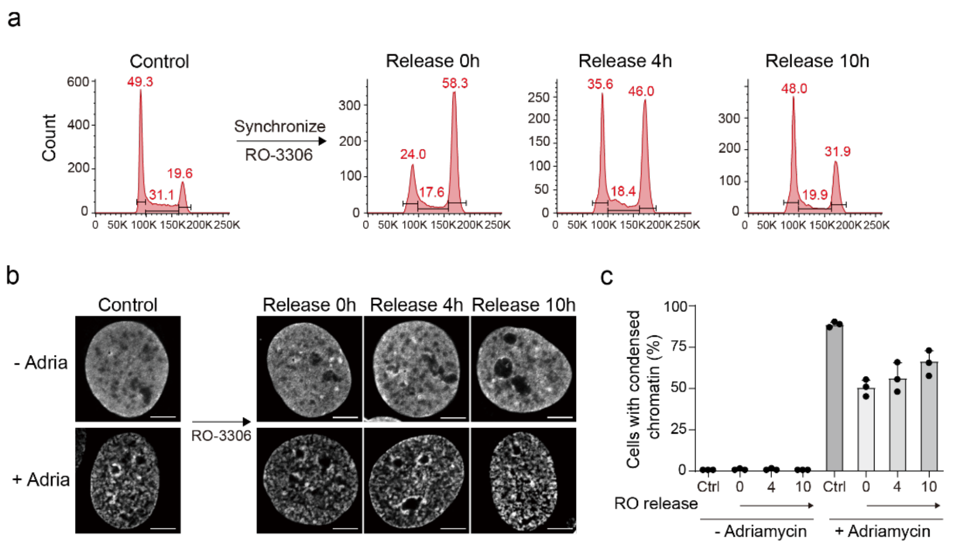

Supplementary Fig. 1. **The correlation between the cell cycle adriamycin-induced condensates**. (a) U2OS cells were synchronized by 9 µM RO-3306 for 18 h before releasing. Cell cycle profiles of 4 and 10 h after releasing from G2/M phase (0 h) were analyzed by FACs after PI staining. (b,c) Representative images and percentages of cells with condensed chromatin at indicated conditions. Before each collection, cells were treated with 1.5 µg/ml adriamycin for 4 h. Scale bar, 10 µm. For each condition in the bar graph, four fields (n=3, each field contains >50 cells) were calculated. Data are presented as means +/- SD.

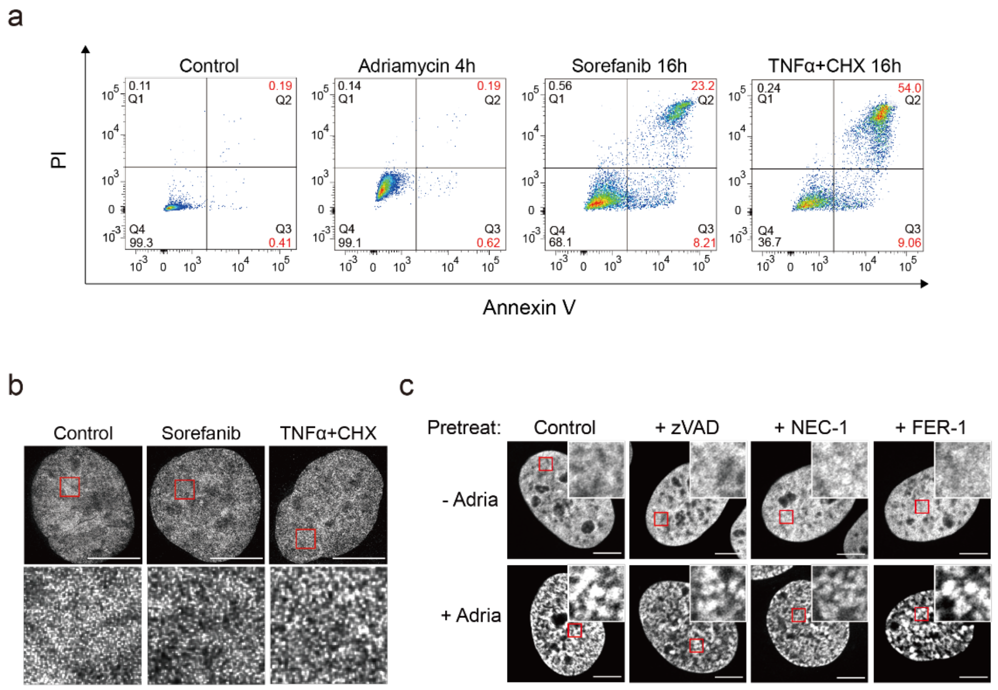

Supplementary Fig. 2. Adriamycin-induced chromatin condensation is independent of apoptosis. (a) Apoptosis analysis indicated that the condition of adriamycin treatment at present study didn't induce early or late apoptosis response as compared with positive controls, sorefanib (15 µM for 16 h) and TNFα/cycloheximide (5 ng/ml / 5 µg/ml for 16 h). (b) Apoptotic cells exhibited distinct chromatin pattern as compared with adriamycin-treated cells. (c) Pre-treatment of inhibitors against apoptosis (zVAD-fmk, 20 µM for 24h), necroptosis (NEC-1, 10 µM for 24h), or ferroptosis (FER-1, 1 µM for 24 h) didn't block adriamycin-induced chromatin condensation. Scale bar, 10 µm. Experiments of a and b were repeated twice, and the experiment of c was repeated three times with similar results.

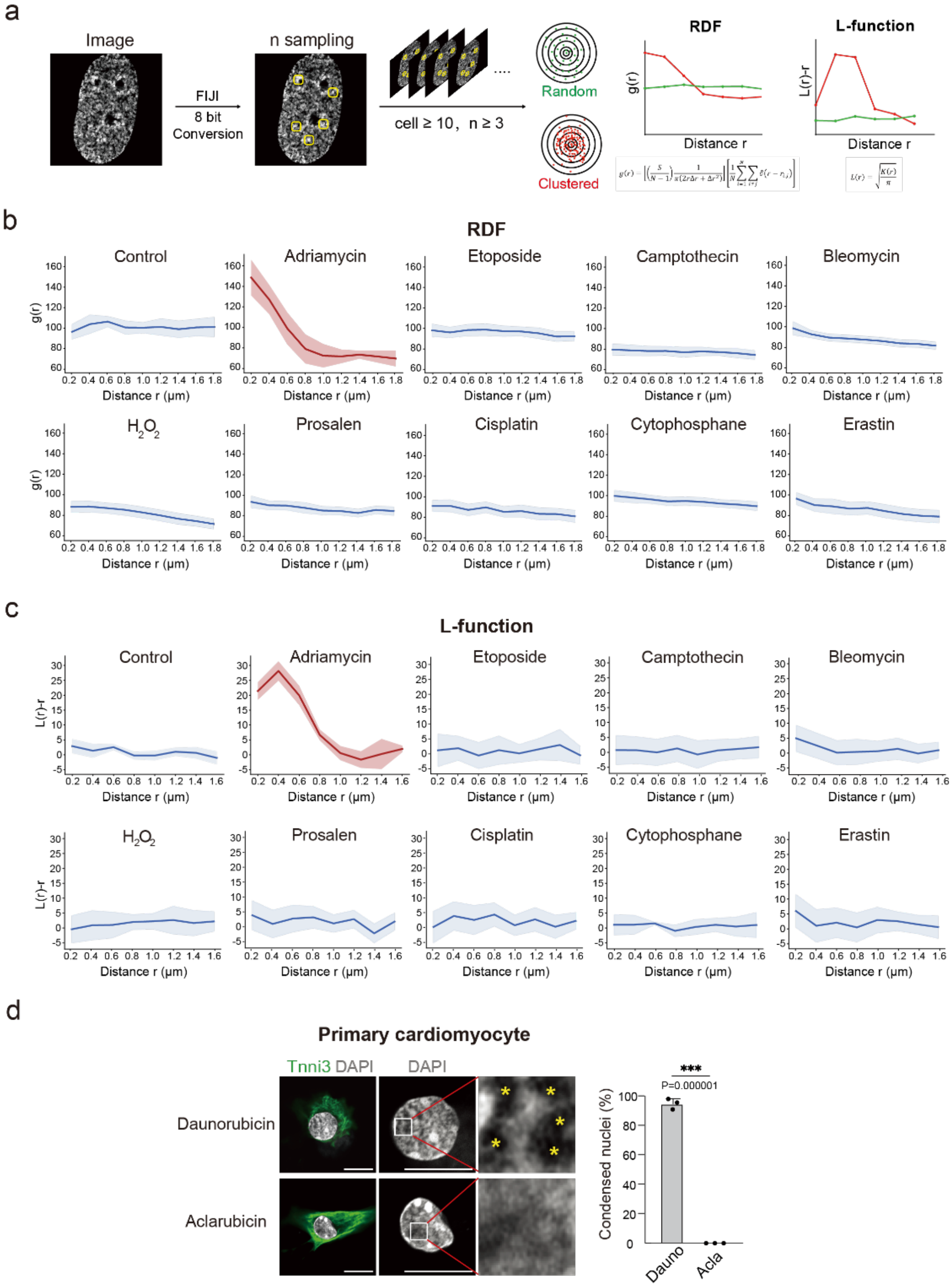

Supplementary Fig. 3. Quantification of the clustering level of chromatin upon the treatment of different drugs. (a) The calculation process of RDF and L-function. The graphs indicate typical curve shapes of chromatins that are randomly-distributed or clustered. Refer to Supplementary Methods for the detail. (b,c) The RDF and L-function of chromatin distribution upon each drug treatment. Related to Fig. 1a. Ribbon plots are used to present means +/- SD (the width of ribbons; n=10 cells). (d) Chromatin structures of daunorubicin (Dauno)- and aclarubicin (Acla)-treated primary cardiomyocytes. Primary cardiomyocytes were treated by 2 μM daunorubicin and 5 μM aclarubicin for 4 h. Cardiomyocytes positive for Tnni3 were examined for their chromatin conformation. For each condition, three fields (n=3) were calculated and totally 42 and 27 cells were counted for each condition For each group in the bar graph, three fields (n=3) were calculated and totally 78, 79, 109, and 79 cells were counted for each group. Data are presented as

means +/- SD. Scale bar, 10 µm. ***, P < 0.001 (two-tailed unpaired t-test).

a

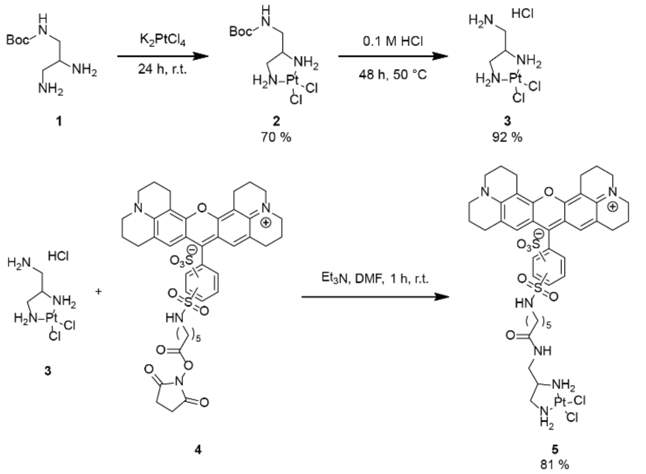

b

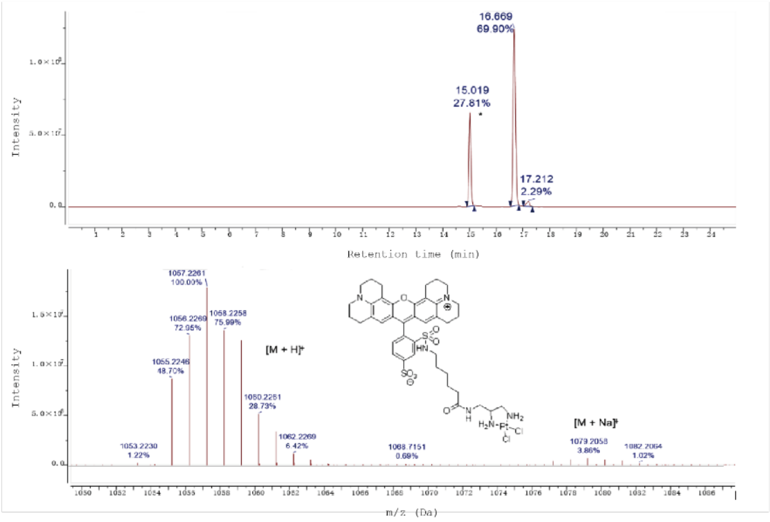

Supplementary Fig. 4. Synthesis and characterization of Texas Red-Cisplatin. (a) Procedure for the preparation of Texas Red-Cisplatin (Compound 5) from K_2PtCl_4, tert-butyl(2,3-diaminopropyl)carbamate (Compound 1), and Texas Red-X succinimidyl ester (Compound 4). Compound 2, N-(tert-Butoxycarbonyl)-1-aminomethyl-1,2-ethylenediamine)dichloroplatinum(II). Compound 3, (1-aminomethyl-1,2-ethylenediamine)dichloroplatinum(II) hydrochloride. Refer to the Supplementary Methods for the synthesis conditions. (b) HPLC-MS analysis of Compound 5. First component with elution time at 15.0 min.

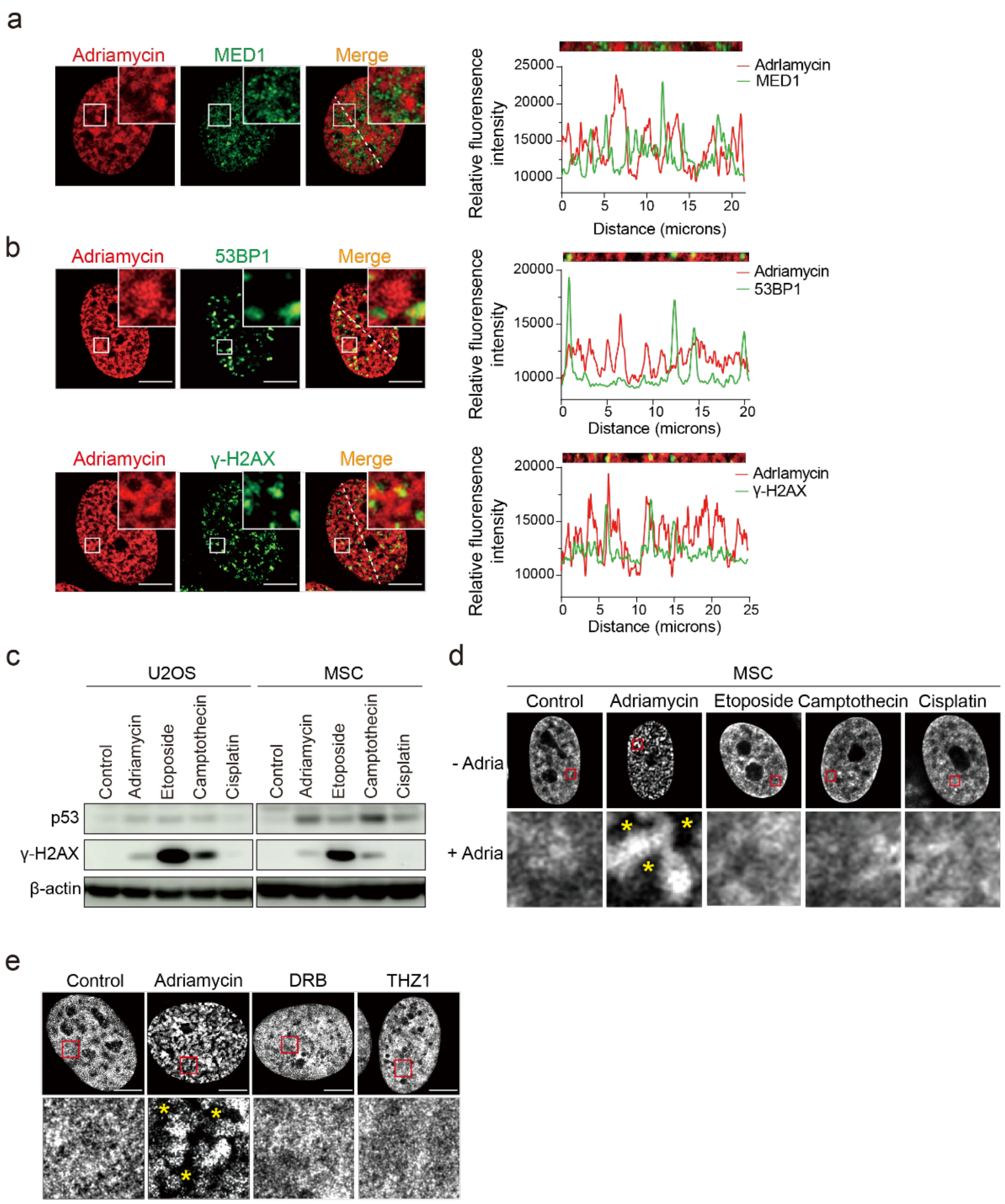

Supplementary Fig. 5. Adriamycin-induced chromatin condensates are irrelevant to transcription- or DNA damage-associated chromatin condensates. (a) Adriamycin-induced chromatin condensates didn't co-localize with Med1 condensates located on enhancers. Scale bar, 10 μm. (b) DNA damage response-associated condensates formed by 53BP1 or γ-H2AX were not co-localized with adriamycin-chromatin condensates. Scale bar, 10 μm. (c,d) The extent of DNA damage response, as measured by H2AX phosphorylation and p53 accumulation in Western blots, was not correlated with the formation of chromatin condensates, which can be exemplified by comparing adriamycin and etoposide treatment. Scale bar, 10 μm. Asterisk, low-density DNA regions. (e) Transcription inhibition by DRB (20 μM, 24 h) or THZ1 (500 μM, 6 h) didn't lead to chromatin condensation at mesoscale. Scale bar, 10 μm. Asterisk, low-density DNA regions. All experiments were repeated at least three times with similar results.

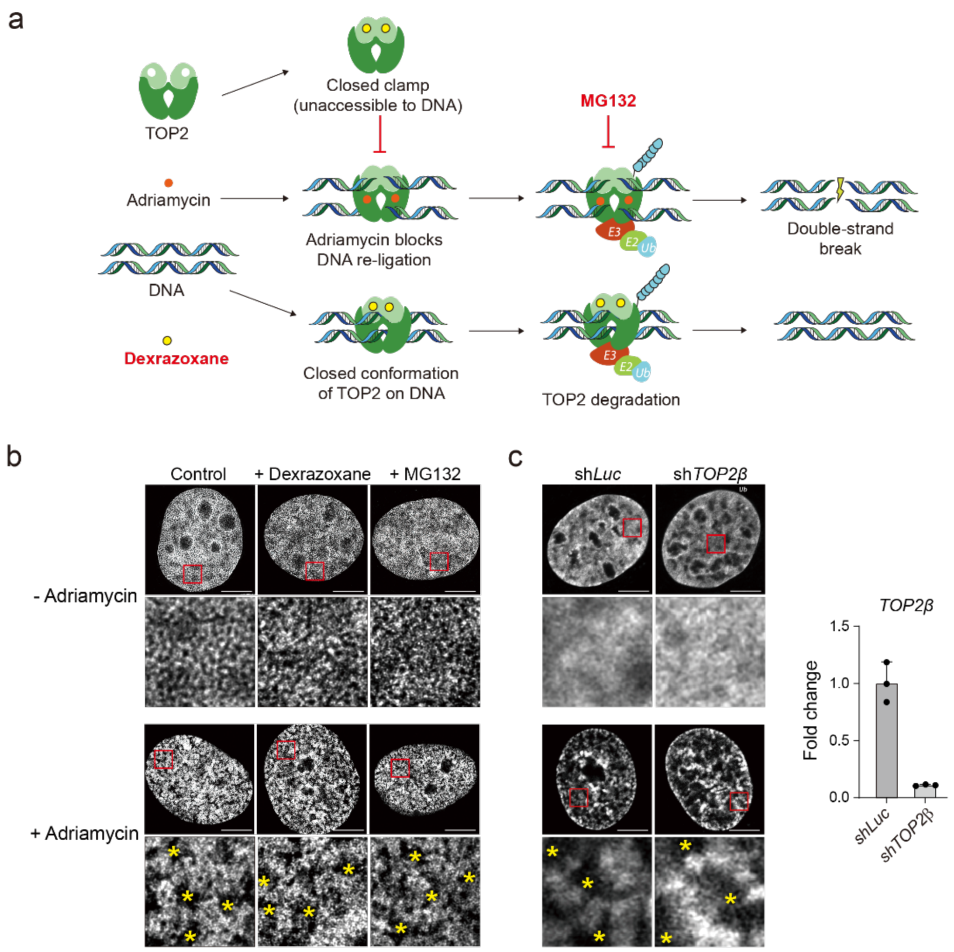

Supplementary Fig. 6. Adriamycin-induced chromatin condensation is independent of TOP2 isozyme. (a) Schematic illustration of the TOP2 poisoning activity of adriamycin. Adriamycin blocks DNA re-ligation of TOP2. After ubiquitin-proteasome mediated degradation, double-strand breaks can be exposed to elicit DNA damage response. The DNA damage response can be blocked by proteosome inhibitor MG132 or dexrazoxane, which induced the closed conformation of TOP2 in the free- or DNA-bound form. The closed conformation of free TOP2 will not be trapped to DNA therefore preventing adriamycin-mediated poisoning. If dexrazoxane binds to DNA-bound TOP2, TOP2 will be depleted by ubiquitin-proteasome system, which also leads to the attenuation of the formation of TOP2-adriamycin cleavable complex. (b) Pretreatment of dexrazoxane (200 µM, 3 h) or MG132 (4 µM, 0.5 h) didn't block the condensation of chromatin induced by adriamycin. Asterisk, low-density DNA regions. The experiment was repeated at least twice with similar results. (c) Knockdown of TOP2β didn't influence the formation of chromatin condensates induced by adriamycin. Asterisk, low-density DNA regions. For the real-time PCR analysis, data are presented as means +/- SD (n=3 for technical replicates).

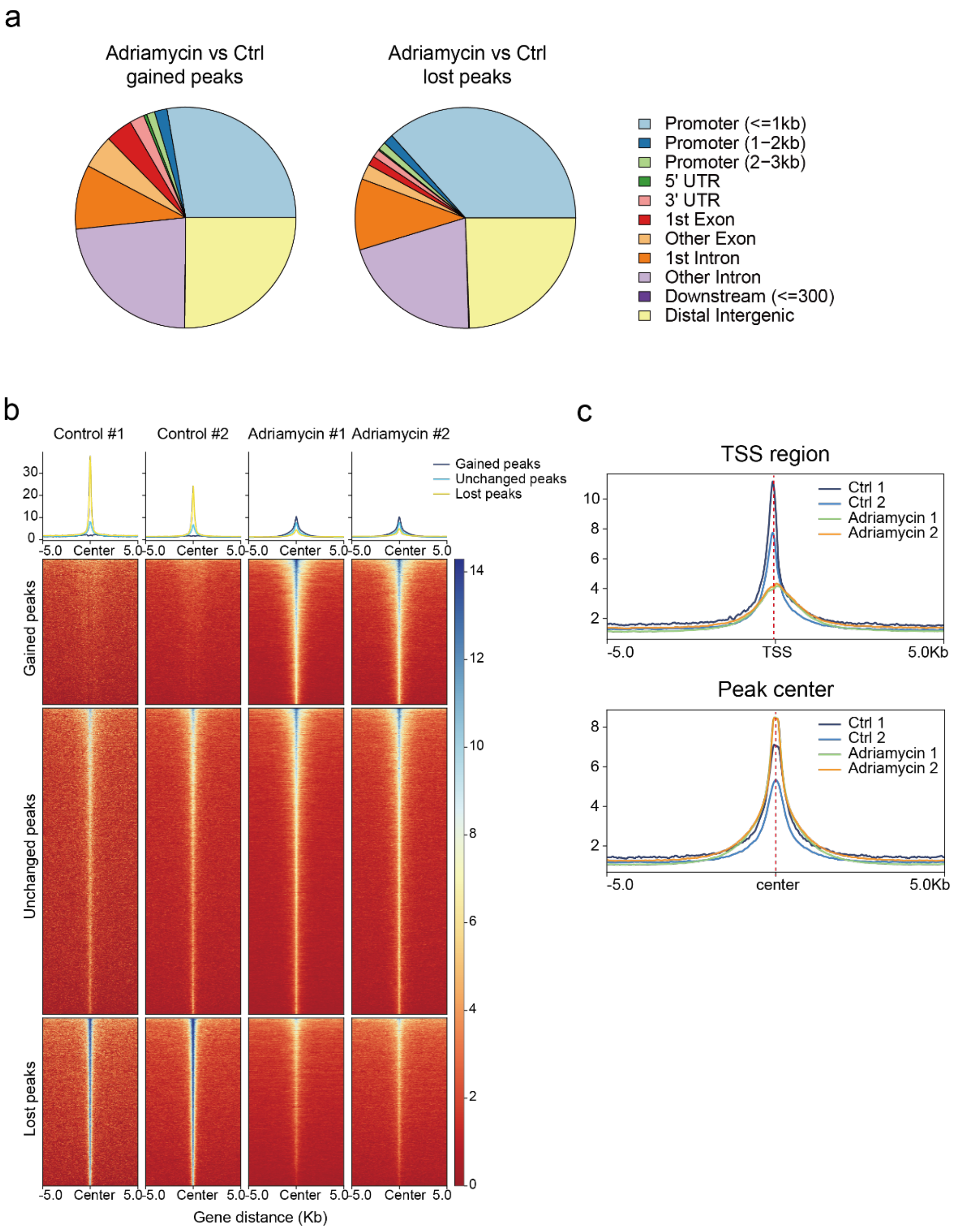

Supplementary Fig. 7. The gain and loss of chromatin accessibility upon adriamycin treatment. (a) Pie charts show the fractions of ATAC-seq peaks associated with different genomic features. The keys show the sum of gained and lost peaks. (b) Averaged line graph and heatmaps show the chromatin accessibility surrounding the center of ATAC-seq peaks in control and adriamycin-treated cells. Note that the extent of loss for peak signals are much more pronounced than the extent of gain. All ATAC-seq peaks shown in the graph are detected in at least one sample. (c) Overlayed line graphs for chromatin accessibility surrounding the TSS (up) or surrounding the center of ATAC-seq peaks (down) in each replicate of control and adriamycin-treated cells. In average, the trend of losing chromatin accessibility upon adriamycin treatment is more significant and consistent surrounding TSS regions.

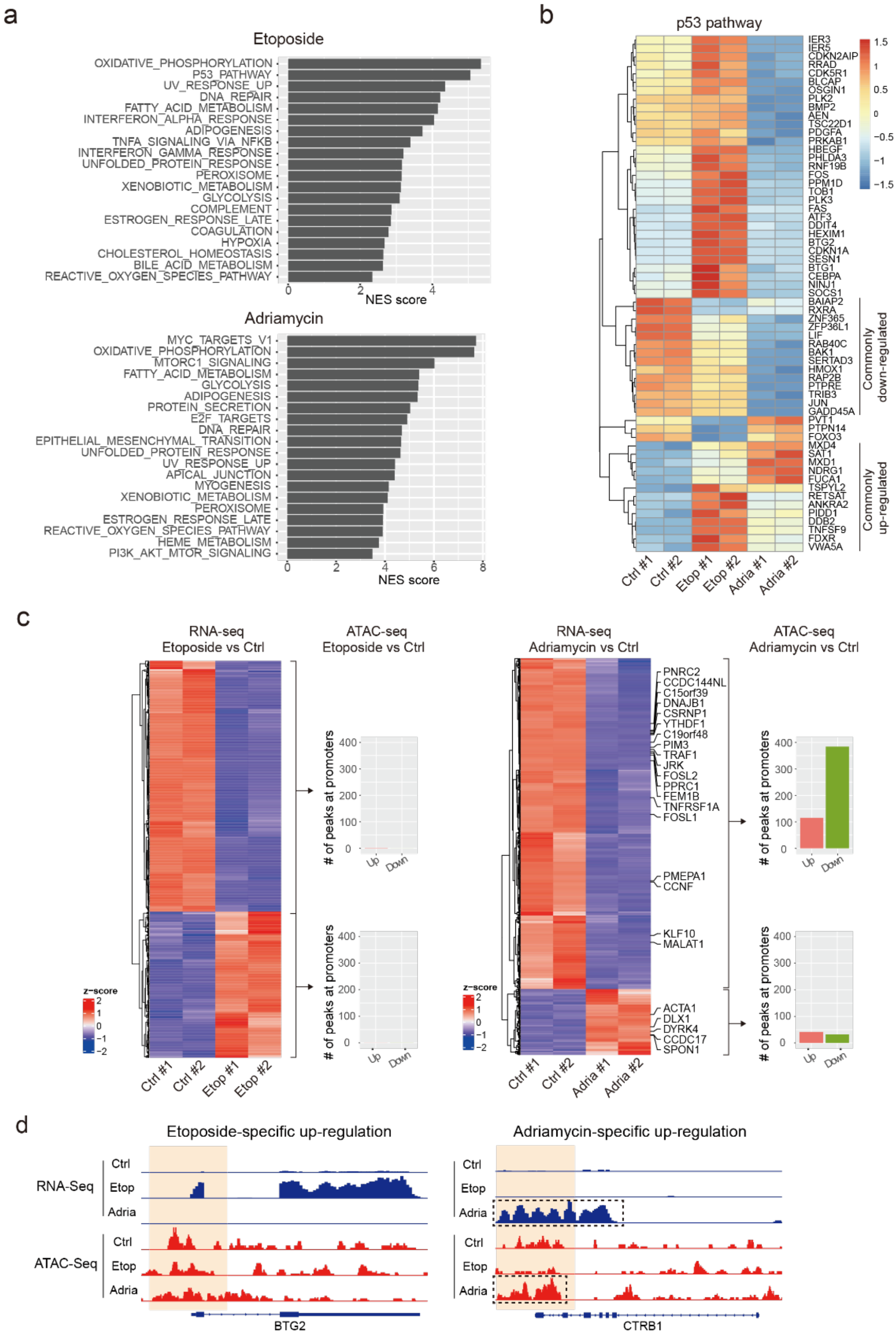

Supplementary Fig. 8. Transcriptomic comparison of etoposide and adriamycin treatment on U2OS cells. (a) GSEA analysis of enriched pathways in etoposide- and adriamycin-treated cells. (b) Expression of p53-regulated genes in etoposide- and adriamycin-treated cells. p53-responsive genes only activated by etoposide may reflect the stronger DNA damage response, as compared with adriamycin. (c) The correlation between transcription level change and chromatin accessibility shifting in etoposide- and adriamycin-treated samples. For etoposide treatment, neither up- or down-regulation of genes were accompanied with changes of chromatin

accessibility. In contrast, genes down-regulated upon adriamycin treatment were correlated with the loss of ATAC-seq peaks. (d) Etoposide-specific activation of genes didn't correspond with the gain of chromatin accessibility surrounding the TSS region, while adriamycin-specific gene activation was correlated with the gain of chromatin accessibility, as exemplified by BTG2 and CTRB1, respectively.

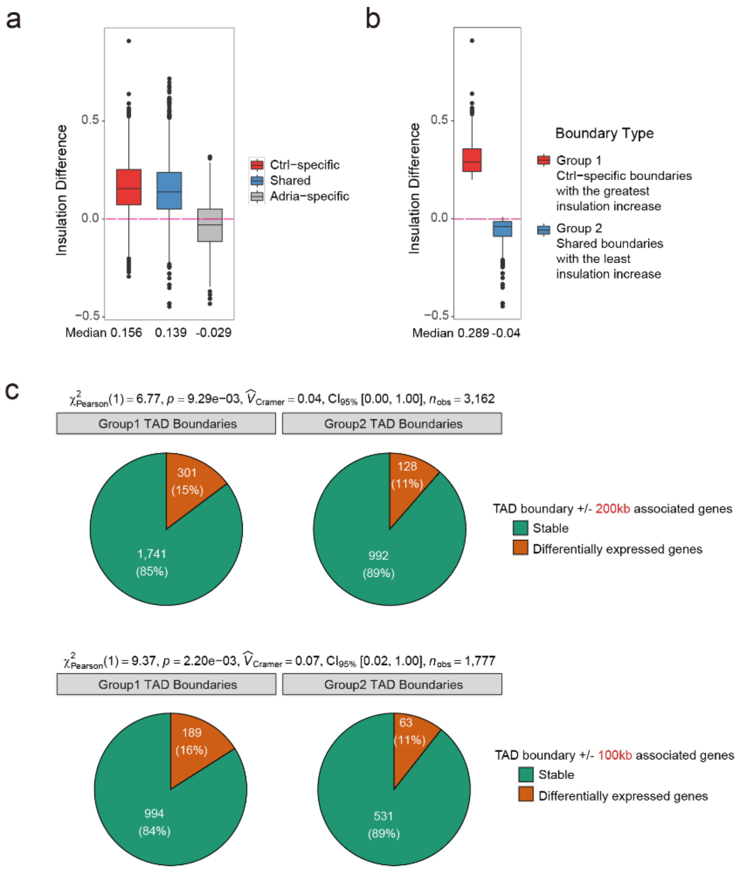

Supplementary Fig. 9. Changes in TAD organization contribute to gene expression shifts. (a) The boxplot summarizes the changes in insulation scores at the control-specific, shared, and adria(adriamycin)-specific TAD boundaries shown in Figure 5E. Increases in insulation scores indicate the weakening of the TAD boundaries. The median of insulation changes for each class of TAD boundaries is indicated below the plot. Note that both the control-specific and the shared TAD boundaries are substantially weakened upon adriamycin treatment. Centerline, median; box limits, upper and lower quartiles; whiskers, 1.5× interquartile range. (b) To assess the relationship between TAD boundary changes and gene expression changes, 375 control-specific TAD boundaries that exhibit an insulation change greater than 0.2 and 322 shared TAD boundaries that exhibit an insulation change less than 0.01 were selected and names as “Group1” and “Group2”. Centerline, median; box limits, upper and lower quartiles; whiskers, 1.5× interquartile range. For (a) and (b), n=2 technical replicates (independently cultured cells were harvested, processed and sequenced independently) for control and adriamycin-treatment conditions. (c) Pie charts illustrates the quantity of differentially expressed genes and stable genes found within each class of TAD boundary with the upstream and downstream expansion of 200kb (up) and 100kb (down). Chi-square tests were conducted to determine the enrichment of differentially expressed genes compared to stably expressed genes within TAD boundaries.

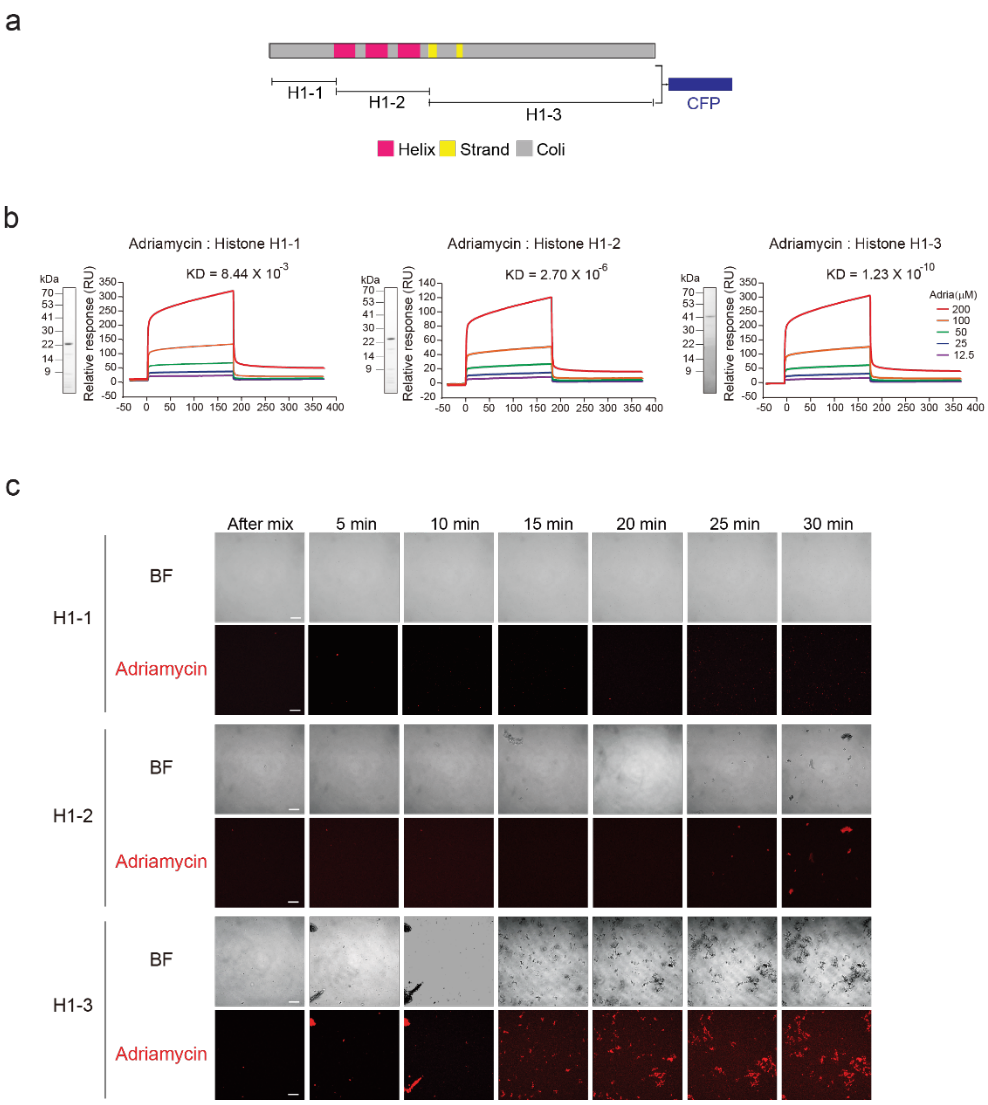

Supplementary Fig. 10. The C-terminal of histone H1 interacts and forms condensates with adriamycin. (a) The structure of H1 was predicted by PSIPRED and dissected to three fragments with 27, 56, and 111 amino acids. Histone H1 fragments fusion with CFP. The fusion proteins were expressed in *E. Coli* and purified by Ni columns. (b) SPR analysis revealed the affinities between adriamycin and H1 fragments, in which the C-terminal of H1 (H1-3) exhibited the highest affinity. (c) *In vitro* condensation of adriamycin and H1 fragments. Mixing adriamycin and H1-3 fusion protein led to the phase separation, forming condensates containing both adriamycin and H1-CFP. Scale bar, 10 μm.

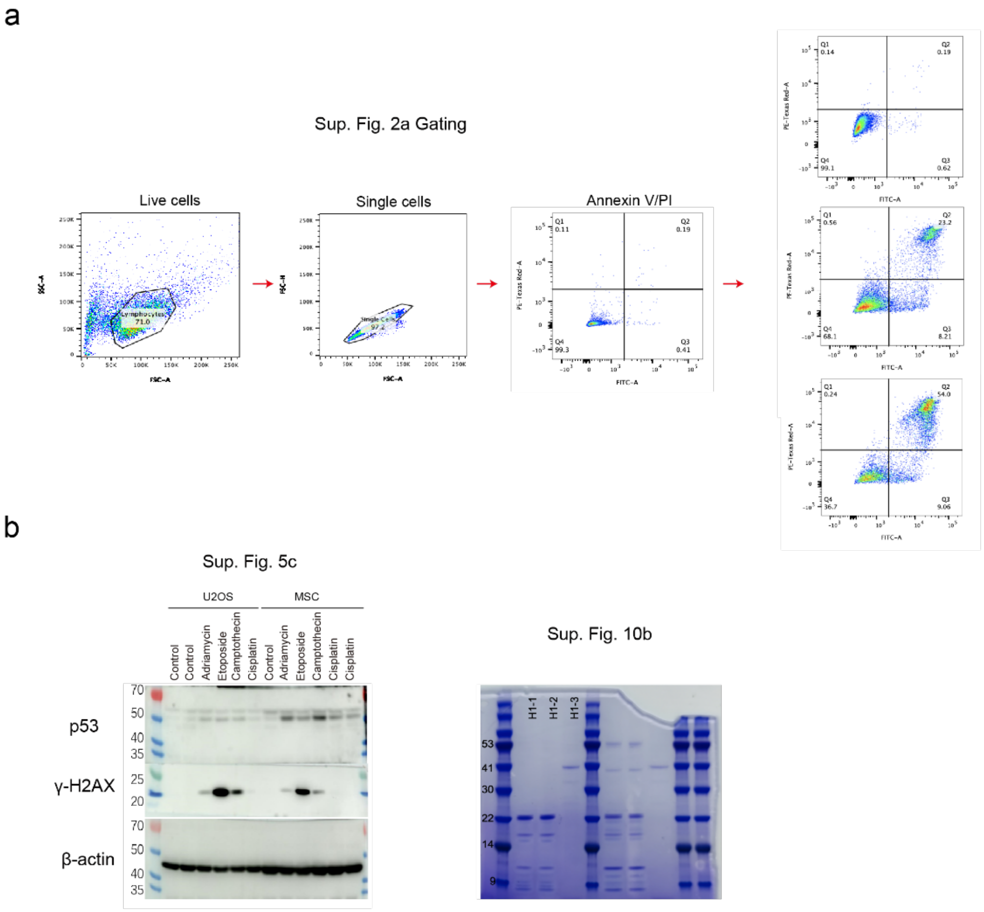

Supplementary Fig. 11. The gating strategy and uncropped blots/gels in Supplementary Figures.

Supplementary Reference

1. Chu, Y.H. *et al.* Systemic Delivery and Biodistribution of Cisplatin in Vivo. *Mol Pharm* **13**, 2677-2682 (2016).
