# Supplementary material for: Chemical-induced phase transition and global conformational reorganization of chromatin
Source: Nat Commun. 2023 Sep 9;14:5556. doi: 10.1038/s41467-023-41340-4 (PMC10492836; doi:10.1038/s41467-023-41340-4)
Supplement: Supplementary file 3 — Description of Additional Supplementary Files [file 41467_2023_41340_MOESM3_ESM.pdf]

Description of Additional Supplementary Files

File name: Supplementary Data 1
Description: List of differentially expressed genes upon etoposide or Adriamycin treatment. Two-tailed P values were calculated by Wald test and the resulting P values were adjusted (Padj) by Benjamini and Hochberg's method for controlling the false discovery rate. Padj<0.01 and |log2(foldchange)| > 1 were set as the threshold for significantly differential expression.

File name: Supplementary Data 2
Description: Association analysis of differentially expressed genes and gain/loss of ATAC-seq peaks. Two-tailed P values were calculated by Wald test and the resulting P values were adjusted (Padj) by Benjamini and Hochberg's method for controlling the false discovery rate. Padj<0.01 and |log2(foldchange)| > 1 were set as the threshold for significantly differential expression.

File name: Supplementary Data 3
Description: List of genes associated with compartment A/B switch. Two-tailed P values were calculated by Wald test and the resulting P values were adjusted (Padj) by Benjamini and Hochberg's method for controlling the false discovery rate. Padj<0.01 and |log2(foldchange)| > 1 were set as the threshold for significantly differential expression.

File name: Supplementary Data 4
Description: List of differentially expressed transposable elements upon etoposide or adriamycin treatment.

File name: Supplementary Movie 1
Description: Live imaging of adriamycin condensate formation in mouse embryonic fibroblasts. Upon the treatment, adriamycin accumulated in the nuclei and formed aggregates with the "large dense" (yellow arrowheads) or the "small fibrous" (white arrowheads) morphologies in mouse embryonic fibroblasts (MEFs). Time-lapse images were collected by Airyscan 2 microscope.

File name: Supplementary Movie 2
Description: FRAP analysis on adriamycin condensates in MEFs. Time-lapse imaging of FRAP analysis shows the material exchange of adriamycin within condensates. Relevant to Fig. 2b.

File name: Supplementary Movie 3
Description: *In vitro* condensation of native chromatin upon adriamycin treatment. Time-lapse microscopic imaging shows adriamycin-induced phase transition of native chromatin. Relevant to Fig. 7b.

File name: Supplementary Movie 4
Description: *In vitro* formation of H1-adriamycin condensates. Time-lapse imaging of the formation of condensates containing both adriamycin (red) and H1-CFP (green). Relevant to Fig. 7e.

File name: Supplementary Movie 5
Description: FRAP analysis on H1-adriamycin condensates in vitro. Time-lapse imaging of the FRAP analysis showing the diffusible property of adriamycin (red) within adriamycin-H1-CFP condensates. Relevant to Fig. 7g.
