# Supplementary material for: Chemical-induced phase transition and global conformational reorganization of chromatin
Source: Nat Commun. 2023 Sep 9;14:5556. doi: 10.1038/s41467-023-41340-4 (PMC10492836; doi:10.1038/s41467-023-41340-4)
Supplement: Supplementary file 13 — Reporting Summary [file 41467_2023_41340_MOESM13_ESM.pdf]

nature portfolio

Corresponding author(s): Qian Bian and Chao-Po Lin

Last updated by author(s): Aug 10, 2023

Reporting Summary

Nature Portfolio wishes to improve the reproducibility of the work that we publish. This form provides structure for consistency and transparency in reporting. For further information on Nature Portfolio policies, see our Editorial Policies and the Editorial Policy Checklist.

Statistics

For all statistical analyses, confirm that the following items are present in the figure legend, table legend, main text, or Methods section.

n/a	Confirmed	
☐	☒	The exact sample size (*n*) for each experimental group/condition, given as a discrete number and unit of measurement
☐	☒	A statement on whether measurements were taken from distinct samples or whether the same sample was measured repeatedly
☐	☒	The statistical test(s) used AND whether they are one- or two-sided<br>*Only common tests should be described solely by name; describe more complex techniques in the Methods section.*
☐	☒	A description of all covariates tested
☐	☒	A description of any assumptions or corrections, such as tests of normality and adjustment for multiple comparisons
☐	☒	A full description of the statistical parameters including central tendency (e.g. means) or other basic estimates (e.g. regression coefficient) AND variation (e.g. standard deviation) or associated estimates of uncertainty (e.g. confidence intervals)
☐	☒	For null hypothesis testing, the test statistic (e.g. *F*, *t*, *r*) with confidence intervals, effect sizes, degrees of freedom and *P* value noted<br>*Give P values as exact values whenever suitable.*
☒	☐	For Bayesian analysis, information on the choice of priors and Markov chain Monte Carlo settings
☒	☐	For hierarchical and complex designs, identification of the appropriate level for tests and full reporting of outcomes
☒	☐	Estimates of effect sizes (e.g. Cohen's *d*, Pearson's *r*), indicating how they were calculated

Our web collection on statistics for biologists contains articles on many of the points above.

Software and code

Policy information about availability of computer code

Data collection	No software was used.
Data analysis	R package

For manuscripts utilizing custom algorithms or software that are central to the research but not yet described in published literature, software must be made available to editors and reviewers. We strongly encourage code deposition in a community repository (e.g. GitHub). See the Nature Portfolio guidelines for submitting code & software for further information.

Data

Policy information about availability of data

All manuscripts must include a data availability statement. This statement should provide the following information, where applicable:

- Accession codes, unique identifiers, or web links for publicly available datasets
- A description of any restrictions on data availability
- For clinical datasets or third party data, please ensure that the statement adheres to our policy

The raw sequencing data of ATAC-Seq, RNA-Seq, and Hi-C generated from this study have been deposited to the GEO database under the accession codes GSE222220, GSE222221, and GSE222637.

Research involving human participants, their data, or biological material

Policy information about studies with human participants or human data. See also policy information about sex, gender (identity/presentation), and sexual orientation and race, ethnicity and racism.

Reporting on sex and gender	N/A
Reporting on race, ethnicity, or other socially relevant groupings	N/A
Population characteristics	N/A
Recruitment	N/A
Ethics oversight	N/A

Note that full information on the approval of the study protocol must also be provided in the manuscript.

Field-specific reporting

Please select the one below that is the best fit for your research. If you are not sure, read the appropriate sections before making your selection.

☒ Life sciences ☐ Behavioural & social sciences ☐ Ecological, evolutionary & environmental sciences

For a reference copy of the document with all sections, see nature.com/documents/nr-reporting-summary-flat.pdf

Life sciences study design

All studies must disclose on these points even when the disclosure is negative.

Sample size	RNA-seq, ATAC-seq and Hi-C data were duplicated for each condition for statistical analysis. Since all experiments were performed on cultured U2OS cells with a short-period (4 hour) of drug treatment, samples in duplicates are predicted to exhibit high reproducibility and sufficient to provide sufficient statistically significance.
Data exclusions	No data were excluded.
Replication	Nearly all experiments presented were repeated at least twice. For key experiments, at least three replications were performed. The detailed information of replication are detailed in individual figure legends.
Randomization	Samples were randomly allocated.
Blinding	Investigators were not blinded due to the objectivity of approaches: most microscopic results (cells and in vitro experiments) are direct phenotype recording. For high-throughput sequencing analyses, same codes are applied to all groups. For some imaging experiments in which statistics are involved, fields were taken randomly and all cells were included for the analysis.

Reporting for specific materials, systems and methods

We require information from authors about some types of materials, experimental systems and methods used in many studies. Here, indicate whether each material, system or method listed is relevant to your study. If you are not sure if a list item applies to your research, read the appropriate section before selecting a response.

Materials & experimental systems

n/a	Involved in the study	
☐	☒	Antibodies
☐	☒	Eukaryotic cell lines
☒	☐	Palaeontology and archaeology
☐	☒	Animals and other organisms
☒	☐	Clinical data
☒	☐	Dual use research of concern
☒	☐	Plants

Methods

n/a	Involved in the study	
☒	☐	ChIP-seq
☐	☒	Flow cytometry
☒	☐	MRI-based neuroimaging

Antibodies

Antibodies used	Anti-Lamin A/C antibody (ABclonal, A19524), 1:100<br>Anti-Tnni3 antibody (ABclonal, A6995), 1:150<br>Anti-Albumin antibody (Life Technologies, A90-134A), 1:150<br>Anti-H3K9me3 antibody (ABclonal, A2360), 1:150<br>Anti-H3K4me3 antibody (Abcam, ab8580), 1:100<br>Anti-H3K27ac antibody (PTM, 116), 1:100<br>Anti-Histone H1 antibody (PTM, 6054), 1:100<br>Anti-MED1 antibody (Abcam, ab64965), 1:200<br>Anti-53BP1 antibody (ABclonal, A5757), 1:200<br>Anti-γ-H2AX antibody (ABclonal, AP0099), 1:200 for IF, 1:1000 for WB<br>Anti-p53 antibody (Santa Cruz Biotech, sc-126), 1:1000<br>Anti-beta-Actin (ABclonal, AC026), 1:1000<br><br>2nd antibodies:<br>HRP goat anti-mouse IgG (ABclonal, AS003), 1: 5000<br>HRP goat anti-rabbit IgG (ABclonal, AS014), 1: 5000<br>Alexa Fluor 647-AffiniPure goat anti-mouse IgG (H+L) (Jackson， 115-605-003) , 1:500<br>Alexa Fluor 647-AffiniPure goat anti-rabbit IgG (H+L) (Jackson， 111-605-003), 1:500
Validation	Lamin A/C antibody: IF, IHC (human); WB (human, mouse, C. elegans); Western Blo (mouse) ; KO validated<br>Tnni3 antibody: WB and IF (mouse, rat)<br>Albumin antibody (Bethyl Laboratories): N/A<br>H3K9me3 antibody: IF (human, mouse), IHC (mouse, rat), WB (human, mouse)<br>H3K4me3 antibody: IF (human), IHC (human), ChIP (human)<br>H3K27ac antibody: WB (human), ICC (human), IP (human)<br>Histone H1 antibody: WB (human), IHC (human), FC (human)<br>MED1 antibody: IHC (human), WB (mouse, human)<br>53BP1 antibody: WB (human), IHC (human)<br>γ-H2AX antioby: WB (human, mouse, rat), IF (mouse, human, rat), IHC (human)<br>p53 antibody: WB (human)<br>beta-Actin antibody: WB (human, mouse, rat), IF (mouse, human, rat), IHC (mouse, human, rat)

Eukaryotic cell lines

Policy information about cell lines and Sex and Gender in Research

Cell line source(s)	National Collection of Authenticated Cell Cultures, China. (U2OS, HCT116, and HeLa)
Authentication	U2OS: STR (CSTR:19375.09.3101HUMSCSP5030)<br>HCT116: STR (CSTR:19375.09.3101HUMSCSP5076)<br>HeLa: STR (CSTR:19375.09.3101HUMSCSP504)
Mycoplasma contamination	All cell lines were negative for mycoplasma.
Commonly misidentified lines<br>(See ICLAC register)	No commonly misidentified cell lines were used in the study.

Animals and other research organisms

Policy information about studies involving animals; ARRIVE guidelines recommended for reporting animal research, and Sex and Gender in Research

Laboratory animals	Wild-type, 6 to 8-week-old C57BL/6 mice
Wild animals	The study did not involve wild animals
Reporting on sex	Sex was not considered in study design since both males and females have to be used for getting pups.
Field-collected samples	The study did not involve samples collected from the field.
Ethics oversight	All mouse experiments were conducted in accordance with Shanghai Institutional Animal Care and Use Committee (IACUC) guidelines and under an approved IACUC protocol of ShanghaiTech University.

Note that full information on the approval of the study protocol must also be provided in the manuscript.

Flow Cytometry

Plots

Confirm that:

☒ The axis labels state the marker and fluorochrome used (e.g. CD4-FITC).

☒ The axis scales are clearly visible. Include numbers along axes only for bottom left plot of group (a 'group' is an analysis of identical markers).

☒ All plots are contour plots with outliers or pseudocolor plots.

☒ A numerical value for number of cells or percentage (with statistics) is provided.

Methodology

Sample preparation	U2OS cells were digested with accutase and combined with suspended cells, washed twice with cold PBS, and resuspended in 1X binding buffer from Annexin V-EGFP/PI apoptosis assay kit (Yeasen, 40303ES20). 100 uL of 1~5 x 106/ml cell suspension was mixed with 5 uL Annexin V-EGFP and incubated in RT for 5 min. After adding 10 L PI staining solution, 400 uL PBS was added before FACs analysis.
Instrument	Fortessa (BD Biosciences)
Software	Flowjo
Cell population abundance	The abundance of cell populations were indicated in the Supplementary Fig. 2
Gating strategy	FSC/SSC gating were used to gate live cells. Annexin V/PI were used to gate early and late apoptotic cells (Supplementary Fig. 2)

☒ Tick this box to confirm that a figure exemplifying the gating strategy is provided in the Supplementary Information.
